# Diagnosis of Frontotemporal Dementia is Often in Non‐Specialist Settings and Complicated by Non‐FTD Dementia and Psychiatric Diagnoses: Patient and Provider Findings from Analysis of a Large US Real‐World Dataset

**DOI:** 10.1002/alz70860_103422

**Published:** 2025-12-23

**Authors:** Jane YC Chan, Carl D Marci, Chiadi U Onyike, David L Cooper

**Affiliations:** ^1^ AviadoBio Ltd., London, United Kingdom; ^2^ OM1, Boston, MA, USA; ^3^ Massachusetts General Hospital and Harvard Medical School, Boston, MA, USA; ^4^ Johns Hopkins University, Baltimore, MD, USA

## Abstract

**Background:**

FTD is a rare young‐onset dementia with prominent behavioral and language symptoms and no approved therapeutics. There are multiple ongoing trials of potentially disease‐modifying treatments for the subset with granulin (GRN) mutations.

It is imperative to understand diagnostic/referral pathways and how brain imaging is used to support the identification of potential opportunities to improve case identification and potential access to future treatments.

We present the results of a retrospective, observational real‐world data (RWD) cohort study characterizing FTD patients and providers. To our knowledge, this is the largest reported RWD study of FTD.

**Method:**

The objective was to identify and characterize patients with FTD, focusing on neurology and mental health (MH) professional consultations (i.e., psychiatrists/psychologists/other professionals), comorbid diagnoses, brain imaging and concomitant medications in a large, multi‐source RWD cohort with electronic health records and insurance claims in over 300 million patient lives in the USA.

The FTD cohort for analysis was defined as records in a period of 3 years before and after the first of 2 identified FTD diagnoses (G31.0 or G31.09) at least 30 days apart (index date).

**Result:**

FTD diagnosis was noted in 41,353 patients with mean age 73.

Among this cohort, few had specialist consultations with either a neurologist (21%) or MH professional (12%). FTD diagnosis was predominantly associated with primary care records; only 17% were associated with neurologists and 3% with MH professionals. Eighty‐two percent had concurrent primary psychiatric disorder including bipolar disorder (60%), depression (49%), anxiety (38%), schizophrenia (16%); 99% had non‐FTD dementia. Brain imaging was infrequent: MRI (22%), CT (29%), either (40%). Concomitant medications mirrored diagnoses: anxiolytics (47%), antidepressants (37%), cholinesterase inhibitor (23%), antipsychotics (23%), and anticonvulsants (21%).

**Conclusion:**

The lack of specialist consultation, limited brain imaging, and high prevalence of concurrent diagnoses of non‐FTD dementias suggests challenges in accurate FTD diagnosis. Further, the high prevalence of psychiatric disorders in absence of MH consultation was unexpected.

Patients with FTD seem to be identified in the community by primary care clinicians, suggesting that future policy and educational efforts may need to focus on supporting appropriate referrals in parallel to patient/public/provider disease‐awareness initiatives.

